# Improved Performance of Magnetic Cross-Linked Lipase Aggregates by Interfacial Activation: A Robust and Magnetically Recyclable Biocatalyst for Transesterification of *Jatropha* Oil

**DOI:** 10.3390/molecules22122157

**Published:** 2017-12-07

**Authors:** Weiwei Zhang, Huixia Yang, Wanyi Liu, Na Wang, Xiaoqi Yu

**Affiliations:** 1 School of Chemistry and Chemical Engineering, National Demonstration Center for Experimental Chemistry Education, Ningxia University, Yinchuan 750021, China; zhangww@nxu.edu.cn (W.Z.); yhx6668297@sina.com (H.Y.); liuwy@nxu.edu.cn (W.L.); 2 Key Laboratory of Green Chemistry Technology, Ministry of Education, College of Chemistry, Sichuan University, Chengdu 610064, China

**Keywords:** lipase, interfacial activation, surfactant, magnetic nanoparticles, cross-linked enzyme aggregates, enzyme immobilization, biodiesel

## Abstract

Lipases are the most widely employed enzymes in commercial industries. The catalytic mechanism of most lipases involves a step called “interfacial activation”. As interfacial activation can lead to a significant increase in catalytic activity, it is of profound importance in developing lipase immobilization methods. To obtain a potential biocatalyst for industrial biodiesel production, an effective strategy for enhancement of catalytic activity and stability of immobilized lipase was developed. This was performed through the combination of interfacial activation with hybrid magnetic cross-linked lipase aggregates. This biocatalyst was investigated for the immobilization of lipase from *Rhizomucor miehei* (RML). Under the optimal conditions, the activity recovery of the surfactant-activated magnetic RML cross-linked enzyme aggregates (CLEAs) was as high as 2058%, with a 20-fold improvement over the free RML. Moreover, the immobilized RML showed excellent catalytic performance for the biodiesel reaction at a yield of 93%, and more importantly, could be easily separated from the reaction mixture by simple magnetic decantation, and retained more than 84% of its initial activities after five instances of reuse. This study provides a new and versatile approach for designing and fabricating immobilized lipase with high activation and stability.

## 1. Introduction

Lipase is one of the most widely used enzymes, and plays an important role in biotechnological and industrial processes including biodiesel, food, detergent, textile, environmental industries, oleochemical industries, as well as in pharmaceutical applications. This is due to their high activity, wide sources and broad range of substrates [1,2]. Under physiological conditions, lipases catalyze the hydrolysis of the ester bonds in the molecules of triglycerides. These substrates are practically insoluble in water, so the reaction is catalyzed at the water–lipid interface, at which most lipases express higher catalytic activity than in aqueous solution. This phenomenon of increased lipase activity at the interface is termed “interfacial activation” [3]. As is well known, the active site of the lipase is covered by a flexible region called a “lid”, which is composed of either one or two amphiphilic a-helices, and blocks substrate access in the closed conformation. In lipases undergoing interfacial activation, the interaction with a drop of oil or a hydrophobic phase can cause opening of the lid to make the active site accessible [4]. As the interfacial activation can lead to a significant increase in catalytic activity, it is of profound importance for all applications of lipases, and should always be taken into account when developing lipase immobilization methods [5].

Previously, different activation methods have been used to immobilize enzymes and can improve their catalytic performance to some extent. These include increasing the hydrophobicity of the supporter for immobilization [6], ion-paired lipases [7], surfactant-coated lipases [8], microemulsions [9], and microemulsion-based organogels [10,11]. As mentioned above, surfactants can activate lipases in different immobilization procedures. An example of these procedures involves coating lipase with surfactant. This is a simple and efficient process demonstrating good stability in a wide range of organic solvents, and can be combined with other immobilization methods [12]. The physical modification of lipases with surfactants can mimic the lipid–water interface, causing opening of the lid to shift the lipase conformational equilibrium toward the open form. This is analogous to the interfacial activation-based molecular bioimprinting [13]. However, there are individual variations among the lipases in surfactant-coating activation. Thus, a concrete analysis should be made when different lipases are used.

Moreover, to fully exploit the technical and economical advantages of lipases, it is recommended to use them in an immobilized form to reduce cost and increase stability of the free lipase. Various methodologies of enzyme immobilization have been established by previous studies including adsorption, ionic binding, covalent modification, entrapment, and encapsulation [14,15]. Efficient immobilization protocols are the result of perfect matching of factors depending on the enzyme, the process, and the support for immobilization [16,17]. In general, lipases can be immobilized using most of the methods developed for enzyme immobilization [18,19]. In the past two decades, cross-linked enzyme aggregates (CLEAs) have provided an innovative, versatile and industrially potent immobilization strategy, arousing extensive attention due to the simplicity of its preparation and robustness of the immobilized enzymes [20]. It has also been successfully applied in the preparation of lipases [21,22]. The CLEA technique appears to involve a superior immobilization method, with prominent advantages over other methods including high volumetric activity, functional stability, multiple recycling potential, and the fact that no purified enzymes are required [23,24,25]. Furthermore, the cross-linking step gives the aggregates a more stabilized structure by generating covalent linkages between enzyme molecules. This renders them permanently insolubilized [26] whilst maintaining their pre-organized superstructure and catalytic activity [27]. 

Despite the simplicity and low cost of preparing CLEAs, a common problem for CLEAs is their irregular shapes and sizes (in the range of 5–100 μm). These factors may lead to slow diffusion of substrates into the CLEAs, or their softness may hinder the process of recovery [28]. These CLEAs are not mechanically resistant, and may require physical support to increase their rigidity for some industrial applications [29]. Although various modifications have been made to further stabilize the CLEAs [30], magnetic bio-separation technology is a promising strategy for the preparation of immobilized enzymes. This technique can be easily performed using an external magnetic field, and provides enhanced stability over repeated uses in continuous bioseparation processes [31,32,33]. Regarding industrial use, attention should also be paid to magnetic carriers because of their low cost and excellent separability. In this work, (3-aminopropyl)triethoxysilane (APTES) was grafted onto the outer surfaces of the magnetic nanoparticles in order to attain the desired properties: high saturation magnetization and sufficient active sites to efficiently improve enzyme loading.

Biodiesel, which consists of a mixture of fatty acid alkyl esters, offers a promising solution for the energy crisis due to its reputation as a sustainable and renewable alternative to fossil fuels. This reputation is based on biodiesels biodegradability, low emission of environmental pollutants and wide range of feedstocks [34]. In light of the current global environment, transesterification of oil feedstock catalyzed by immobilized lipase has been regarded as one of the most promising techniques for preparing biodiesel. This is due to their high activity and selectivity for alcoholysis of triglycerides under mild reaction conditions [35]. Therefore, potential industrially relevant biocatalysts are in high demand. However, biocatalysts that are capable of both increased enzyme loading and improved enzymatic stability are rare.

In this study, a strategy integrating surfactant-activated magnetic cross-linked enzyme aggregates was developed and investigated for the immobilization of lipase from *Rhizomucor miehei* (RML). This was performed in order to combine advantageous properties such as high specific enzyme activity, stable open-lid conformation of lipase, ease of separation and recovery, and sufficient active sites, into a single system for lipase immobilization. The APTES functionalized magnetic supports were used as a rigid and multifunctional cross-linking reagent, able to reduce the mobility of the lipase lid and produce immobilized lipases with a stabilized open form. Some specific characteristics of the immobilized RML were examined further, including optimization of the immobilization conditions and the stability of the immobilized enzyme. Moreover, the prepared immobilized lipase was utilized to catalyze transesterification for biodiesel production. 

## 2. Results and Discussion

### 2.1. Characterization of the Immobilized Lipase

**Figure 1:**
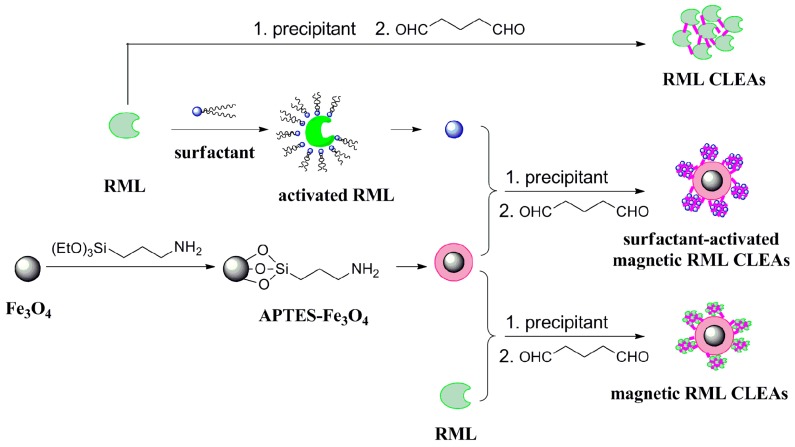
Schematic illustration of the immobilization process to produce *Rhizomucor miehei* (RML) cross-linked enzyme aggregates (CLEAs), magnetic RML CLEAs, and surfactant-activated magnetic RML CLEAs.

In this study, a strategy integrating interfacial activation, magnetic nanoparticles, and lipase CLEAs immobilization was developed in order to combine advantageous properties into a single system for enzyme immobilization. These properties include ease of separation and recovery, and sufficient enzyme loading. As illustrated in Figure 1, aminopropyl-functionalized magnetic nanoparticles were readily prepared and subsequently employed for the immobilization of lipase from *Rhizomucor miehei*. 

**Figure 2:**
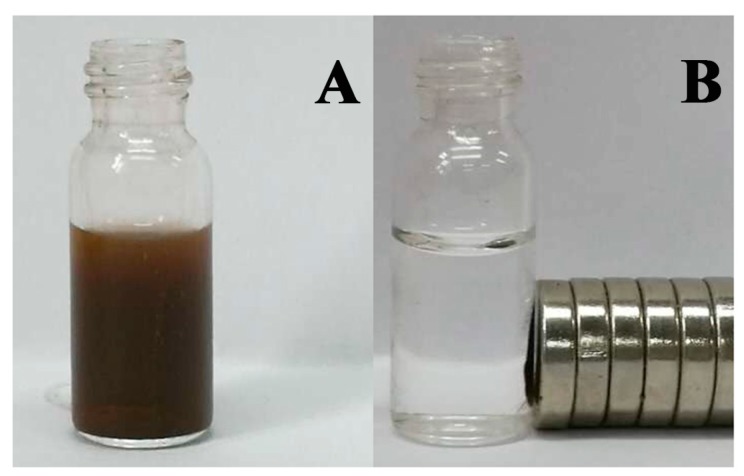
Schematic of the simple magnetic separation of surfactant-activated magnetic RML CLEAs: (**A**) immobilized lipase dispersed in reaction mixture; (**B**) immobilized lipase collected by an external magnet.

As can be seen in Figure 2, all of the magnetic immobilized RMLs exhibited rapid separation from the reaction mixture using a magnet. Once the magnetic field has been removed, the immobilized enzyme can be easily dispersed by simple shaking. This can provide an easy and efficient way to separate immobilized enzyme from a suspension system. The successful immobilization of lipase onto the APTES-Fe_3_O_4_ nanoparticles was identified by FT-IR spectroscopy and scanning electron microscopy.

**Figure 3:**
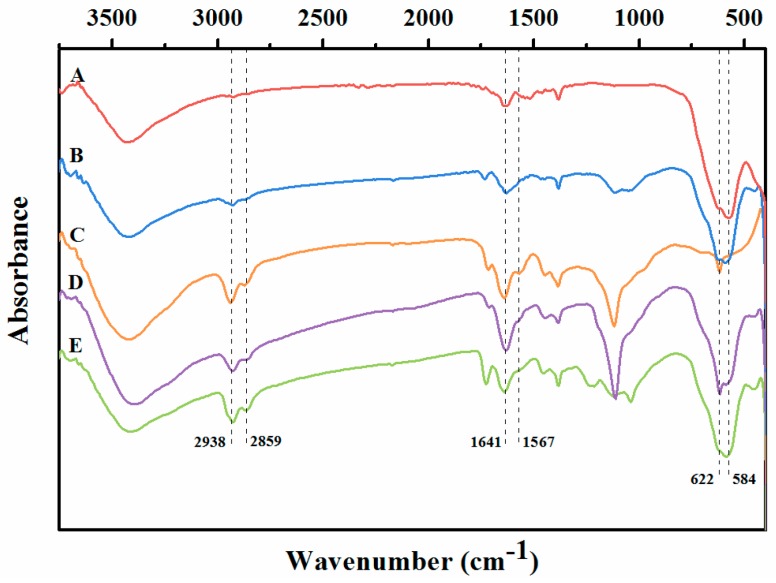
The FT-IR spectrum of (**A**) Fe_3_O_4_ nanoparticles; (**B**) (3-aminopropyl)triethoxysilane (APTES)-functionalized Fe_3_O_4_ nanoparticles; (**C**) RML CLEAs; (**D**) magnetic RML CLEAs; (**E**) AOT-activated magnetic RML CLEAs. (Sodium bis-2-(ethylhexyl) sulfosuccinate, AOT).

Figure 3 shows the comparative FT-IR spectra of the magnetic core, APTES-functionalized Fe_3_O_4_ particles, RML CLEAs, and magnetic RML CLEAs. In the spectrum of magnetic RML CLEAs, new characteristic peaks at 584 cm^−1^ correspond to the Fe-O vibrations of the magnetite core. This can be ascribed to the successful binding of Fe_3_O_4_ particles. As compared with the magnetic nanoparticles, the immobilized lipase displayed additional IR bands situated at 1641 cm^−1^ and 1567 cm^−1^, which represent the amide I (the stretching vibrations of C=O groups) and amide II (N-H bending and C-N stretching) bands [36] of RML, respectively. Furthermore, after the lipase loading, it was noted that new bands at 2938 cm^−1^ and 2859 cm^−1^ appeared for magnetic RML CLEAs, which was reasonably attributed to C-H stretching vibrations, which also indicated the successful loading of lipase.

**Figure 4:**
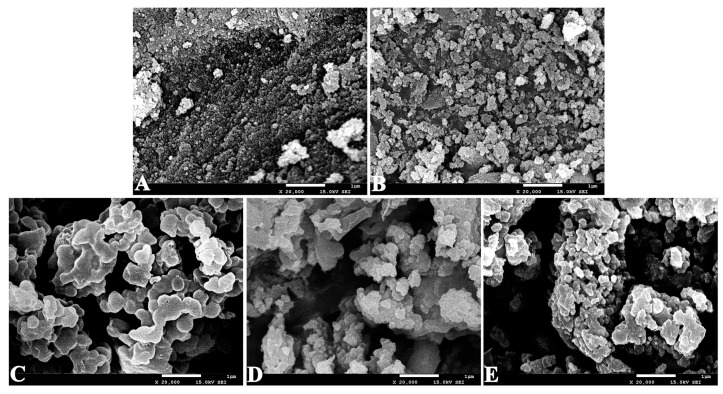
SEM images of (**A**) Fe_3_O_4_ nanoparticles; (**B**) APTES-functionalized Fe_3_O_4_ nanoparticles; (**C**) RML CLEAs; (**D**) magnetic RML CLEAs; (**E**) AOT-activated magnetic RML CLEAs.

SEM studies monitored the morphological changes of the magnetic nanoparticles and different immobilized RML preparations, as illustrated in Figure 4. The typical SEM image of the bare Fe_3_O_4_ particles in Figure 4A indicates that the aggregates of Fe_3_O_4_ particles exhibit an obvious spherical shape, and present a clearly tight structure. This is due to the strong magnetic dipole–dipole interactions between Fe_3_O_4_ nanoparticles. Figure 4B shows representative SEM images of the aminopropyl-functionalized Fe_3_O_4_ particles. As displayed in this figure, APTES-Fe_3_O_4_ nanoparticles showed a rough surface morphology, with an approximate size of 30–50 nm, implying that the modification of ATPES on the surface of the Fe_3_O_4_ nanoparticles could weaken the aggregation of the magnetic nanoparticles. As indicated in Figure 4C, RML CLEAs display a smooth surface, and their lengths ranged from 300 to 1000 nm. This indicates that the surface of CLEAs became more compact, perhaps improving enzyme rigidity for further applications. However, this also limits the diffusion of substrate to access lipase. When modified Fe_3_O_4_ particles were added before lipase precipitation, the magnetic particles could act as cores during lipase precipitation, whilst also cross-linking with lipase. Therefore, this procedure could not only prevent the formation of secondary particles (Figure 4D) but also improve the stability of the immobilized enzymes. It is noteworthy that the formed APTES-Fe_3_O_4_ nanoparticles not only display magnetic behaviours, but also have large active surface available for lipase immobilization. After coating with AOT, RML CLEAs were uniformly dispersed on the magnetic particles (Figure 4E). These observations suggested that the coating layer of surfactants could prevent the formation of large aggregates. Therefore, the specific surface area of the RML CLEAs could be effectively improved.

**Figure 5:**
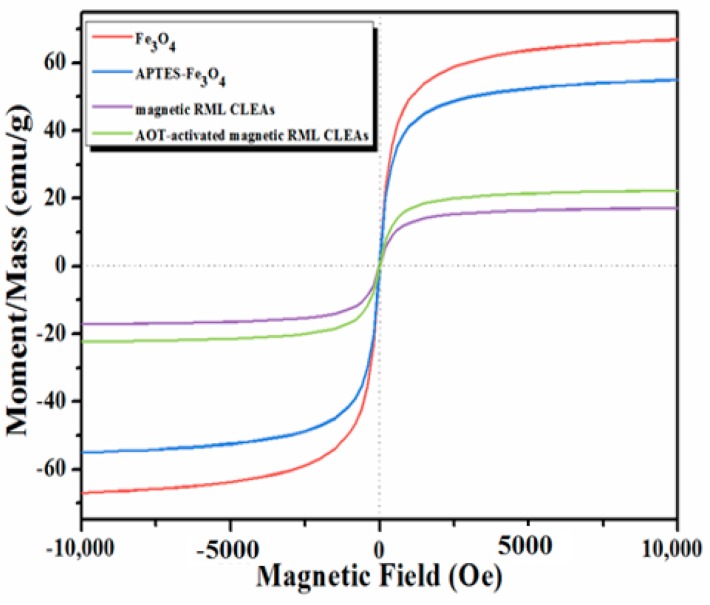
Magnetic hysteresis loops of Fe_3_O_4_ nanoparticles, APTES-functionalized Fe_3_O_4_ nanoparticles, magnetic RML CLEAs and AOT-activated magnetic RML CLEAs.

In addition, the magnetic measurements of the magnetic nanoparticles and different immobilized RML preparations were displayed in Figure 5. As illustrated in Figure 5, the magnetization curves exhibit no hysteresis, indicating the superparamagnetic character of all these samples. The saturation magnetization of immobilized RML preparations is lower than that of magnetic nanoparticles, owing to the presence of lipase-loading. Generally, these properties allow for easy and rapid separation of the AOT-activated magnetic RML CLEAs from the reaction mixture using a magnet.

### 2.2. Optimization of Magnetic RML CLEAs Preparation Parameters

#### 2.2.1. Effect of Precipitant

**Figure 6:**
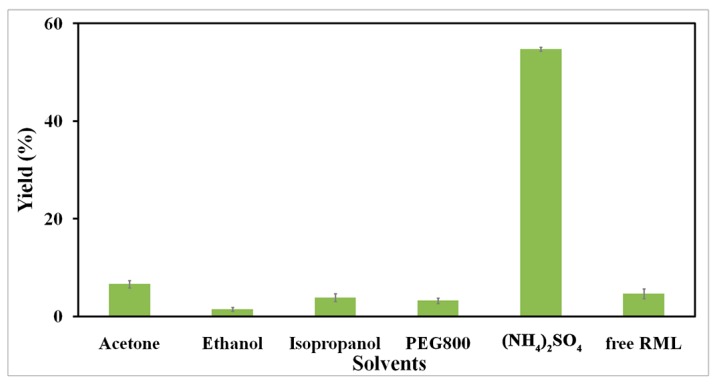
Effect of precipitant type on the activities of magnetic RML CLEAs.

The foremost step in preparation of CLEAs is precipitation. The characteristics of the precipitant that allow it to achieve the maximum activity recovery in aggregation can differ from one lipase to another. This is due to the diversity of biochemical and structural properties of the enzymes [37]. Lipases from different microbial sources display different glycosylated surfaces. This may result in different aggregation, packing and configuring of the protein molecules with different precipitating reagents [26]. To limit maximum enzyme activity in magnetic RML CLEA preparation, five commonly used protein precipitating agents were investigated, including organic solvents (acetone, ethanol, isopropanol), polyethylene glycol (PEG 800) and ammonium sulfate. As shown in Figure 6, different types of precipitant exhibited varying degrees of activity for CLEAs. In addition, significantly higher activity of RML CLEAs was retained in saturated ammonium sulfate, and can therefore be used as the precipitant of choice for further studies.

#### 2.2.2. Effect of Glutaraldehyde Concentration

**Figure 7:**
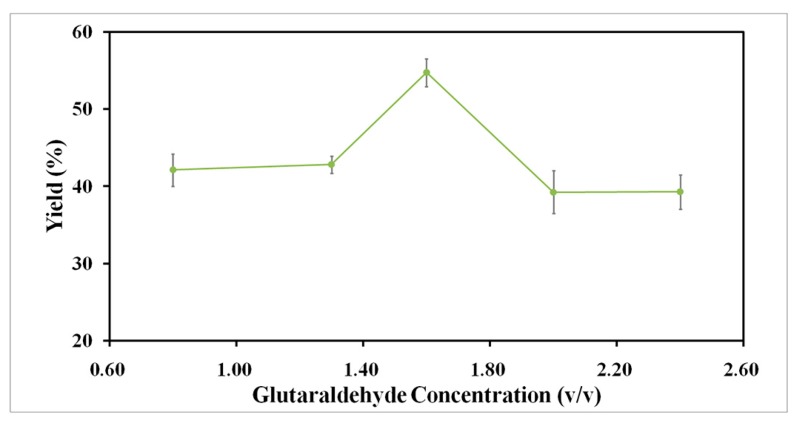
Effect of glutaraldehyde concentration on the on the activities of magnetic RML CLEAs.

Cross-linking of the precipitated RML and amino functionalized magnetic nanoparticles is the second step in the preparation of magnetic RML CLEAs. In this step, lipase aggregates are permanently packed into an active and insoluble form. Glutaraldehyde is a powerful cross-linker that is widely used in the design of biocatalysts because it is cost effective and readily available in commercial quantities [38]. Since the enzyme activity, stability and particle size of the resulting magnetic CLEAs are deeply affected by the ratio of cross-linker to enzyme [39], magnetic RML CLEAs were prepared using different concentrations of glutaraldehyde. As shown in Figure 7, the transesterification yield of the magnetic RML CLEAs was enhanced using increasing glutaraldehyde concentrations up to 1.6% (*v*/*v*). However, the transesterification yield decreased with the addition of excessive amounts of glutaraldehyde. This result implies that excessive glutaraldehyde might result in increasing enzyme rigidity due to a more intensive cross-linking. This restricts enzyme flexibility and influences the active site availability, thus decreasing the activity of magnetic RML CLEAs.

#### 2.2.3. Effect of Lipase-to-Nanoparticle Ratio

**Figure 8:**
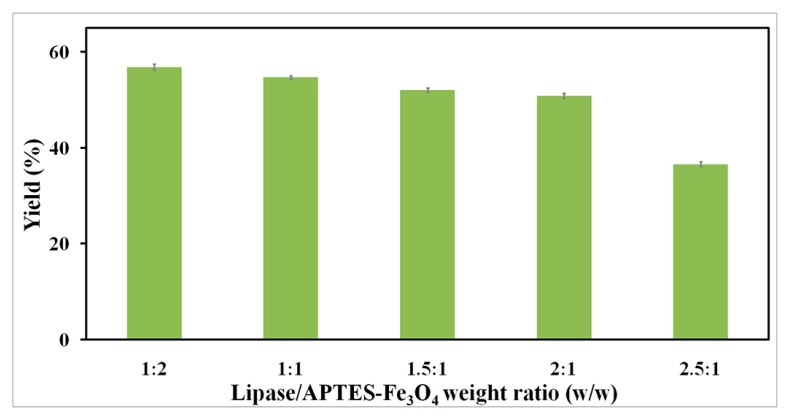
Effect of the lipase-to-nanoparticle ratio on the activity of magnetic RML CLEAs.

To achieve effective immobilization, the transesterification yield was determined with the lipase-to-nanoparticle weight ratio varying from 1:2 to 2.5:1 during magnetic CLEA preparation. Accordingly, as shown in Figure 8, the transesterification yields decreased as the weight ratio of lipase and nanoparticle increased. A higher concentration of enzyme naturally leads to a higher enzyme loading amount. However, the loading efficiency decreases with increasing enzyme concentration. In order to maximize efficient utilization of magnetic nanoparticles, the optimal lipase-to-nanoparticle weight ratio of 1:1 was selected as the most appropriate option for magnetic RML CLEA preparation.

### 2.3. Surfactants Activated Lipase Magnetic CLEAs

RML is a stable and widely used lipase with a molecular size of 31,600 Da and an isoelectric point (pI) of 3.8. The structure of RML revealed a Ser-His-Asp trypsin-like catalytic triad with an active serine buried under a 15 amino acid long “lid” [40]. In the closed conformation, the lid covers the active site, thereby blocking the access of substrate molecules [3]. Interaction with a hydrophobic phase can cause opening of the lid, making the active site accessible. Moreover, considering that RML tends to form bimolecular aggregates with reduced activity, and the presence of detergents may break these dimers, the use of surfactant-coated procedure may be a good option to create interfacially activated and monomeric RML molecules.

**Figure 9:**
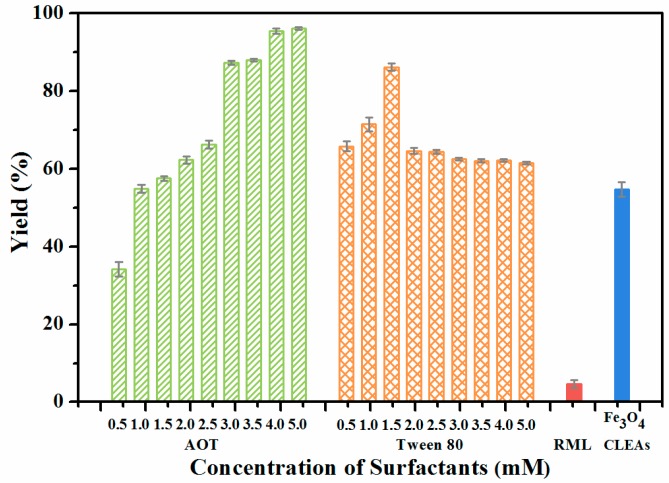
Effect of activation of different surfactants on activity of magnetic RML CLEAs.

To fix the lipase in an “open conformation”, an integrated strategy of interfacial activation and covalent cross-linking immobilization was developed, and surfactant-coated RML was sequentially used for magnetic CLEAs preparation. As shown in Figure 9, the presence of AOT or Tween 80 during the magnetic CLEAs preparation promotes the activity of magnetic RML CLEAs to varying degrees compared to the free RML and RML CLEAs. When Tween 80 was used, magnetic RML CLEAs were more active at lower concentrations (0.5 to 1.5 mM). This decreased with increasing concentrations, suggesting that Tween 80 could not provide effective activation of RML. Meanwhile, the activity of magnetic RML CLEAs significantly increased with the addition of AOT, indicating that AOT was the optimal amphiphile for the activation of RML, and that the optimum concentration was 4.0 mM.

Under the optimal conditions, the activity recovery of the AOT-activated magnetic RML CLEAs was as high as 2058%, with a 20-fold improvement over the free RML. As is well known, the mechanism for improving the activity and stability of the immobilized lipase is extremely complicated. The significant increase in enzyme activity is not only related to the immobilization method, but also to the unique characteristics of lipase, as well as the carrier properties. The active centers of most lipases are covered by a so-called “lid” structure, which controls access of the substrates to the active site. The secondary structure of the lipase probably changes during immobilization, and the lid might be opened to some extent for the substrates. This would provide easier access by the substrates, leading to an increase in lipase activity. Molecular dynamics simulations of the RML lid postulated that, among other interactions, Arg86 within the lid stabilized the open-lid conformation of the protein through multiple hydrogen bonds to the protein surface [41]. Moreover, the electrostatic interactions of Arg86 play an important role in terms of both the intrinsic stability and displacement of the lid. This action occurs through enhancement of hinge mobility in a high dielectric medium [42]. Therefore, the interaction between the anionic head group in the AOT molecule and the positively charged Arg86 residue might be able to shift the lipase conformational equilibrium toward the open form, and stabilize the open forms of RML. Furthermore, the coating of anionic AOT could provide a better micro-aqueous environment for lipase molecules. It may also cause greater dispersion and decrease agglomeration by exerting a smaller effect on the three-dimensional structure of negatively charged lipase. These coated molecules tend to offer improved enzyme activity. Furthermore, APTES functionalized magnetic nanoparticles can provide a larger surface area and sufficient active sites, resulting in higher immobilization efficiency and larger contact area between the substrate and the enzyme. This can effectively decrease mass transfer resistance, leading to higher enzyme activity.

### 2.4. Application in Biodiesel Production

#### 2.4.1. Effect of Organic Solvents on Biodiesel Production

To estimate the practical application of immobilized RML, AOT-activated magnetic RML CLEAs were also investigated as efficient nano-biocatalysts for enzymatic transesterification in production of biodiesel from *jatropha* oil. A series of experiments was performed to determine the optimal conditions for fatty acid methyl ester (FAME) production. This result suggested that activation of surfactants before immobilization could anchor the lipase in an activated state, thus keeping it in an active form for subsequent application.

**Figure 10:**
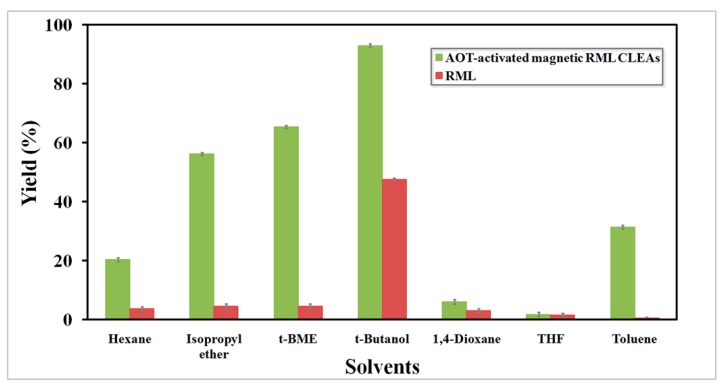
Effect of different solvents on AOT-activated magnetic RML CLEAs catalyzed biodiesel production from *jatropha* oil.

The reaction medium plays a significant role in maintaining the catalytic activity and stability of an enzyme. In order to select the suitable medium for the lipase reaction, the production of FAMEs was performed in various conventional organic solvents at 40 °C and the results compared to the results obtained from free RML. Interestingly, AOT-activated magnetic RML CLEAs produced yields greater than 20% in all of the anhydrous solvents (except for toxic 1,4-dioxane and THF), whereas little product was observed when free RML was used (Figure 10). The solubility of *jatropha* oil is much higher in *tert*-butanol, *t*-BME and isopropyl ether, resulting in a decrease in mass transfer resistance of the substrates and substantial enhancement of FAME yields. Meanwhile, the hydrophilic THF and 1,4-dioxane could more easily strip away the essential water bound to the lipase by participating in non-covalent solvent protein interactions, resulting in distortion of their active conformation and thus loss of their activity. This substantial enhancement suggested that surfactant activation can be combined with covalent cross-linking to anchor the lipase in an open active form, thus enhancing their stability in polar solvents. As depicted in Figure 9, the highest yield of FAMEs was obtained in *tert*-butanol for both free (47%) and immobilized RML (93%). It has been reported that biodiesel production in the presence of *tert*-butanol improves the solubility of methanol and reduces the inhibitory effect of glycerol, therefore improving the yield of reaction [43].

#### 2.4.2. Effect of Temperatures on Biodiesel Production

**Figure 11:**
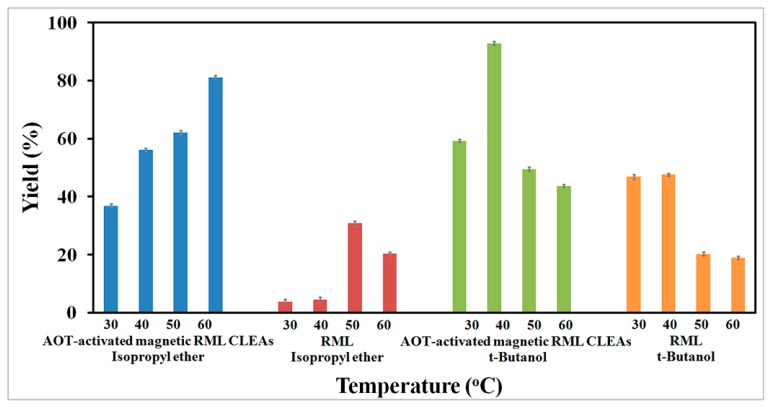
Effect of temperatures on AOT-activated magnetic RML CLEAs-catalyzed biodiesel production from *jatropha* oil in isopropyl ether and tert-butanol.

As temperature is an important factor to be considered in the enzymatic preparation of biodiesel, biodiesel production was carrying out in isopropyl ether and *tert*-butanol at different temperatures (30–60 °C). According to Figure 11, AOT-activated magnetic RML CLEAs were more active in the range of 30–60 °C than free RML, either in isopropyl ether or *tert*-butanol. Increasing lipase stability after immobilization can be attributed to rigidification of the lipase structure, which prevents unfolding of the protein. As the temperature gradually increases from 30 to 60 °C, there is a rise in the biodiesel yield catalyzed by immobilized RML in isopropyl ether. However, the optimum temperature of the free form was found to be 50 °C in the same medium. Meanwhile, when *tert*-butanol is used as the solvent, the optimum temperature for AOT-activated magnetic RML CLEAs was found to be 40 °C, with biodiesel yields of about 93%. Based on the results, 40 °C was selected for further studies.

#### 2.4.3. Reusability

**Figure 12:**
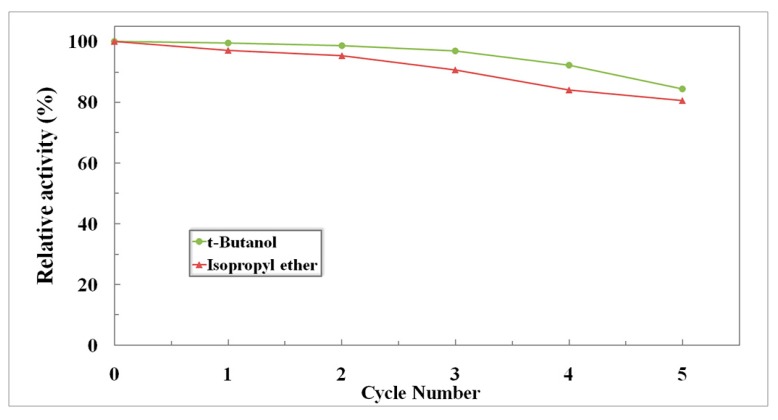
Reuse of AOT-activated magnetic RML CLEAs for biodiesel production from *jatropha* oil in isopropyl ether and tert-butanol.

One of objectives of using an immobilized enzyme is to design a more efficient biocatalyst that can easily be recovered and reused. Magnetic immobilization offers the benefits of easy separation of enzyme from the reaction system, allowing for consequent reuse of enzyme, as well as the potential to run a continuous process. In this work, the reusability of AOT-activated magnetic RML CLEAs was evaluated in consecutive batches of the biodiesel reaction in isopropyl ether and *tert*-butanol, respectively. After completion of each batch, the immobilized enzyme was recovered by magnetic separation and washed with solvent to prepare it for the subsequent batch. The initial activity prior to the first recovery was taken as 100%, and the activity in the subsequent reactions was calculated accordingly. As presented in Figure 12, AOT-activated magnetic RML CLEAs performed well with repetition up to 5 cycles, with 84% and 81% of activity retained in isopropyl ether and *tert*-butanol respectively. This implies that the immobilized RML possesses excellent long-term operational stability. It is noteworthy that the AOT-activated magnetic RML CLEAs displayed a fast response (20 s) to an external magnetic field, which indicated that this level of saturation magnetization would be adequate for the separation of the immobilized enzyme.

## 3. Materials and Methods 

### 3.1. Materials

Lipase from *Rhizomucor miehei* (solution) and methyl ester standards (methyl palmitate, methyl stearate, methyl oleate, methyl linoleate, and methyl tridecanoate) were purchased from Sigma-Aldrich (St. Louis, MO, USA). Substrates containing 3-aminopropyl triethoxysilane (APTES), glutaraldehyde (25%, *v*/*v*) and 2-phenyl ethanol (>98%, CP) were purchased from Aladdin (shanghai, China). Sodium bis-2-(ethylhexyl) sulfosuccinate (AOT) were obtained from Acros (Pittsburgh, PA, USA). *Jatropha* oil was obtained from Yanyuan County, Sichuan Province, China. All other chemicals were of analytical or chromatographical grades and used without further purification. Double distilled water was employed throughout the experiments.

### 3.2. Functionalization of Magnetic Nanoparticles

Magnetic Fe_3_O_4_ nanoparticles were prepared by the conventional co-precipitation method. In a typical procedure, 1.20 g (6.0 mmol) of FeCl_2_·4H_2_O and 3.25 g (12.0 mmol) of FeCl_3_·6H_2_O were dissolved in 50 mL deionized water under nitrogen at room temperature. Then, 10 ml 25% ammonia solution was added dropwise under a nitrogen atmosphere with vigorous stirring, resulting in the immediate formation of black precipitates. After aging in the mother solution for 3 h, the obtained magnetic particles were washed several times with deionized water until neutral, and dried at 100 °C for 2 h. To obtain amino functionalized magnetic particles, magnetic nanoparticles were suspended in a solution composed of 2.5 mL methanol, 100 μL of 3-aminopropyl trimetoxysilane and 25 μL of deionozed water. The resulting mixture was homogenized by ultrasonication for 30 min. Then glycerol (1.5 mL) was introduced into the solution and allowed to reflux at 90 °C for 6 h with maximum mechanical agitation. Finally, the APTES-Fe_3_O_4_ nanoparticles were washed several times with methanol and deionized water, magnetically separated, and subsequently lyophilized prior to use.

### 3.3. Immobilization of Lipase

#### 3.3.1. Preparation of RML CLEAs

RML CLEAs were prepared according to the procedure reported by Jia et al. [33]. Firstly, 5 mL of saturated ammonium sulphate solution was added into 1 mL of RML solution (10 mg/mL, 0.1 M phosphate buffer, pH 7.0), and stirred for 30 min at 4 °C. After precipitation of RML, glutaraldehyde was added slowly to the final concentration of 1.6% *v*/*v,* and stirred for 3 h at 30 °C. After cross-linking, 4 mL of phosphate buffer (0.1 M, pH 7.0) was used to dilute the suspension, and the mixture was then centrifuged at 10,000 rpm for 5 min. The resultant precipitates were washed thrice with phosphate buffer and deionized water, lyophilized and finally stored at 4 °C.

#### 3.3.2. Preparation of Magnetic RML CLEAs

Magnetic RML CLEAs were produced by mixing with 10 mg of APTES-Fe_3_O_4_ nanoparticles and 1 mL of RML solution (10 mg/mL, 0.1 M phosphate buffer, pH 7.0) and shaken for 15 min at 30 °C. Then 5 mL of precipitant was added with stirring at 4 °C for 30 min. After precipitation, glutaraldehyde was added dropwise into the suspension, and stirred for 3 h at 30 °C. All subsequent procedures were the same as those for the RML CLEAs preparation. 

#### 3.3.3. Preparation of Surfactants-Activated Magnetic RML CLEAs 

The surfactant-activated RML was prepared using anionic Aerosol OT (Sodium bis-2-(ethylhexyl) sulfosuccinate, AOT) and nonionic Tween 80 at various concentrations. Firstly, 1 mL of RML solution and an appropriate amount of surfactant were mixed and stirred at 4 °C for 30 min. After incubation for 24 h at 4 °C, the suspended solution was sequentially used for magnetic RML CLEAs preparation. Free RML, RML CLEAs and magnetic RML CLEAs served as controls. 

### 3.4. Assay of Immobilized RML

#### 3.4.1. Activity Assay

The activities of free lipase and different immobilized preparations were determined by the transesterification reaction of 2-phenyl ethanol and vinyl acetate. Firstly, 10 mg of 2-phenylethanol was mixed with 1 mL of vinyl acetate. To start the reaction, 10 mg of RML (the initial amount of RML CLEAs and magnetic RML CLEAs was 10 mg) was added, and the mixture was reacted in a temperature-controlled shaker at 30 °C, 220 rpm for 24 h. The reaction was terminated by the isolation of immobilized lipase using a magnet. The samples were withdrawn from the reaction medium at regular intervals and analyzed by high-performance liquid chromatography (HPLC). All experiments were repeated at least three times.

#### 3.4.2. Biodiesel Production

To evaluate the effectiveness of immobilized lipase catalyzed *jatropha* oil bioconversion, the transesterification experiments were conducted as follows. In a typical experiment, the reaction was performed in a 10 mL screw-capped vessel containing 2.0 mL solvent, 0.5 g of *jatropha* oil and anhydrous methanol, at oil-to-methanol molar ratio of 1:3. The reaction was initiated by the addition of 20 mg RML (the initial amount of RML CLEAs and magnetic RML CLEAs was 20 mg). Then the mixture was incubated in a temperature-controlled shaker at 40 °C, 220 rpm for 48 h. All biodiesel reactions were performed in dried solvents without any water added. Aliquots (20 μL) of the reaction mixture were withdrawn at various time intervals throughout the reaction time, and then diluted with *n*-hexane for GC analysis. Effects of solvents and reaction temperatures on the biodiesel production were studied by single-factor experiment design. 

#### 3.4.3. Reusability

The enzyme reuse was evaluated in the transesterification reaction of *jatropha* oil with methanol. Upon completion of one cycle, the immobilized derivative was separated by a permanent magnet, washed thrice with solvent, and then re-suspended in a fresh reaction mixture for the next catalytic cycle. The biodiesel yield of the first reaction was set as 100% and the ester yield in the subsequent reactions was calculated accordingly.

#### 3.4.4. HPLC Analysis

HPLC was conducted with Waters Associates equipment (Waters 2695 with 2998 Photodiode Array Detector). A C18 column was used in the HPLC experiments with MeOH/water = 80:20 (*v*/*v*). The wavelength of the UV detector was set at 254 nm, the column temperature was maintained at 30 °C during the assays, and the flow rate was 0.8 mL/min. 

#### 3.4.5. GC Analysis

The fatty acid methyl ester (FAMEs) content of the reaction mixture was quantified using a Fuli9790 gas chromatography (Fuli, China) equipped with an AT.SE-54 column (30 m × 0.25 mm × 0.33 μm). Nitrogen was used as the carrier gas at a constant flow of 1.5 mL/min. The column temperature was held at 160 °C for 2 min, and raised at 10 °C/min to 240 °C, where it was then maintained for 10 min. The temperatures of the injector and the detector were set at 280 and 270 °C, respectively. By comparing the retention times and peak areas of standard fatty acid methyl ester peaks, the total quantities of biodiesel in the reaction mixtures were calculated.

### 3.5. Characterization

#### 3.5.1. FT-IR

The Fourier transform infrared (FT-IR) analysis was carried out using Shimadzu FTIR-4200 spectrometer in the range of 400 to 4000 cm^−1^. A standard KBr pellet technique was applied for the sample preparation.

#### 3.5.2. SEM

The morphology of the particle surface was observed using a scanning electron microscope (SEM, JSM 7500F; JEOL, Tokyo, Japan). The capsule was freeze-dried and coated with gold before it was analyzed.

#### 3.5.3. Magnetization Measurements

The magnetic properties of the magnetic nanoparticles and different immobilized RML preparations were detected at room temperature using a vibrating sample magnetometer (VSM, MicroSense EZ9, Lowell, MA, USA).

## 4. Conclusions

In the present study, lipase from *Rhizomucor miehei* was activated with surfactant and used in preparation of magnetic CLEAs in order to obtain active hybrid catalysts for the transesterification of *jatropha* oil with methanol to produce fatty acid methyl esters (FAMEs). Since the coating of surfactant fixes RML in its open form, the activity of the final derivatives increased remarkably compared to the free RML. The nature of the precipitant and surfactant, as well as the concentration of glutaraldehyde, are the key factors affecting the immobilization of RML. Under the optimal conditions, the AOT-activated magnetic RML CLEAs displayed 20-fold improvement in transesterification activity compared with the free RML. This increased activity is attributed to the enhanced interfacial activation of lipase due to the interaction between lipase and appropriate surfactant coating. Moreover, the immobilized RML showed excellent catalytic performance for the biodiesel reaction and, more importantly, could be easily separated from the reaction mixture by simple magnetic decantation. It also retained more than 84% of its initial activity after five instances of reuse. The interfacial activation favors the open conformation of lipase, and consequently the substrate availability for the immobilized enzyme. Therefore, the combination of interfacial activation, magnetic nanoparticles, and cross-linked enzyme aggregates in lipase immobilization has high potential for industrial application, and presents an attractive potential process for other lipase immobilization in the future.
